# A Four-Year Diagnostic Journey to Acute Intermittent Porphyria in a 19-Year-Old Woman: Lessons on Recognizing Neurovisceral Clues

**DOI:** 10.7759/cureus.114827

**Published:** 2026-08-20

**Authors:** Dilshad Rezwan, S.R. Dhinakharan

**Affiliations:** 1 Emergency Medicine, Luton and Dunstable University Hospital, Luton, GBR

**Keywords:** abdominal pain, acute intermittent, diagnostic delay, hydroxymethylbilane synthase/deficiency, porphyrias, seizures

## Abstract

Acute intermittent porphyria (AIP) is a rare autosomal dominant metabolic disorder caused by hydroxymethylbilane synthase (HMBS) deficiency, resulting in the accumulation of neurotoxic porphyrin precursors. Its rarity and overlap with psychiatric, neurological, and gastrointestinal clinical features frequently lead to diagnostic delays and substantial morbidity. Therefore, we report the case of a 19-year-old woman who presented to a UK tertiary hospital with recurrent tonic-clonic seizures and severe episodes of abdominal pain over a four-year period. After initial investigations excluded cardiac and epileptic causes, she was diagnosed with pseudoseizures and referred to a psychiatric facility because no conclusive epileptiform activity was detected on electroencephalography (EEG). Multiple gastrointestinal diagnostic investigations, including gastric emptying studies, were unsuccessful in identifying an underlying cause. Following postoperative deterioration characterized by nausea, vomiting, and seizures, porphyria screening was initiated. Fecal total porphyrins were elevated at 72.3 nmol/g (reference range, 0-49.9 nmol/g), raising strong biochemical suspicion of an acute hepatic porphyria and prompting specialist referral, while plasma and urinary porphyrin levels remained within normal limits. The patient was referred to a specialist porphyria clinic for genetic confirmation and management. This case illustrates the diagnostic complexity of AIP, in which overlapping neurological and psychiatric features can frequently result in misdiagnosis, and demonstrates that fecal porphyrin elevation can provide valuable complementary biochemical evidence when other investigations are inconclusive, particularly when testing is performed outside an acute episode. AIP should therefore be considered in young women with recurrent unexplained seizures and abdominal pain, especially when standard investigations, including urinary aminolevulinic acid (ALA) and porphobilinogen (PBG), are unremarkable, as normal urinary ALA and PBG levels measured outside an acute attack do not exclude AIP. Early metabolic screening, including fecal porphyrin analysis, may prevent years of morbidity and facilitate timely specialist referral.

## Introduction

Acute intermittent porphyria (AIP) is the most common of the acute hepatic porphyrias and is inherited in an autosomal dominant pattern, resulting from a deficiency of hydroxymethylbilane synthase (HMBS) [1]. This enzymatic defect leads to the accumulation of highly neurotoxic porphyrin precursors, principally delta-aminolevulinic acid (ALA) and porphobilinogen (PBG) [2]. These precursors, particularly ALA, are thought to be directly toxic to the autonomic, peripheral, and central nervous systems, accounting for the neurovisceral manifestations of an acute attack. AIP usually remains latent but can be precipitated by specific physiological stressors, such as hormonal changes, intercurrent infections, prolonged fasting, or exposure to porphyrogenic drugs [3]. AIP predominantly affects women of reproductive age (15-45 years); therefore, accurate and timely diagnosis is particularly important in this population [4]. The recommended first-line biochemical investigations during a symptomatic acute attack are urinary PBG and ALA. Plasma and fecal porphyrin measurements are complementary investigations, primarily used to help distinguish between the different acute hepatic porphyrias.

The diagnosis is inherently challenging because of the nonspecific clinical presentation, which can involve multiple systems, including severe abdominal pain, neurological dysfunction (seizures and paresis), and neuropsychiatric symptoms [5]. A high index of clinical suspicion is therefore essential, as the symptoms can mimic more common psychiatric, neurological, or gastrointestinal disorders. Delayed diagnosis can have severe consequences, including acute complications such as flaccid paralysis, respiratory failure, and permanent neurological deficits [6], as well as chronic morbidity, including systemic arterial hypertension, chronic kidney disease, and hepatocellular carcinoma [7].

Here, we report the case of a 19-year-old woman with recurrent seizures and episodic abdominal pain over a four-year period who was eventually diagnosed with AIP.

This case serves as a clinical warning regarding the consequences of delayed metabolic screening in young women presenting with unexplained recurrent neurovisceral symptoms and highlights the complementary role of fecal porphyrin analysis during the inter-attack period.

## Case presentation

A 19-year-old woman presented to a UK tertiary hospital with a four-year history of recurrent tonic-clonic seizures and episodic abdominal pain. At the onset of symptoms (year 1), she experienced three generalized tonic-clonic seizures over approximately three hours, each preceded by a prodrome of nausea, palpitations, and chest pain. An electrocardiogram (ECG) demonstrated a prolonged corrected QT interval (QTc) of 470 ms (reference range: <460 ms). However, detailed pediatric cardiology assessment, including 24-hour ambulatory cardiac monitoring and event recording, yielded entirely normal results, effectively excluding a primary cardiac disorder.

Over the following year (years 1-2), the patient continued to experience episodes of collapse and paroxysmal involuntary movements. Sequential electroencephalography (EEG) studies were performed. A standard EEG was nonspecific, a sleep-deprived video-EEG demonstrated prominent beta activity without definitive epileptiform discharges, and a 23-hour ambulatory EEG indicated only a “potential predisposition to epilepsy.” In the absence of confirmatory electrophysiological evidence, she was diagnosed with pseudoseizures following multidisciplinary review at the pediatric seizure clinic and a complex case meeting. She was discharged with a formal recommendation for psychological support through the Child and Adolescent Mental Health Services (CAMHS).

Concurrently, and persisting over years 2-4, the patient experienced recurrent episodes of abdominal pain accompanied by intractable nausea and vomiting, prompting multiple hospital admissions and gastrointestinal evaluations. Gastroparesis was excluded by a nuclear medicine gastric emptying study. A laparoscopic appendicectomy was subsequently performed for suspected acute appendicitis; histopathological examination confirmed early acute appendicitis, with no evidence of granuloma or neoplasia. Postoperatively, the patient presented again within one week with bilious vomiting, abdominal pain, lethargy, and generalized weakness. Abdominal imaging excluded obstruction, and she was managed conservatively under a pelvic inflammatory disease protocol following gynecological review. This diagnosis was made on clinical grounds in the absence of a confirmed alternative cause and, given the subsequent diagnostic trajectory, likely represented a further misattribution of the underlying porphyric symptoms.

Four years after symptom onset, the patient presented to the Emergency Department with an acute seizure. Cranial CT and MRI were normal, excluding a structural etiology. Given the pattern of unexplained, prolonged abdominal symptoms in the context of recurrent seizure-like activity, the gastroenterology team subsequently initiated porphyria screening. Fecal total porphyrins were elevated at 72.3 nmol/g (reference range: 0-49.9 nmol/g), providing the key biochemical finding that prompted specialist referral to a porphyria service and further evaluation. Urinary ALA (2.4 µmol/mmol creatinine; reference range: <5 µmol/mmol creatinine) and PBG (0.8 µmol/mmol creatinine; reference range: <1.5 µmol/mmol creatinine) were within normal limits, consistent with testing performed outside an acute attack. Red cell HMBS activity was 38.6 U, a low-normal value relative to the local laboratory reference range. Although this finding can be seen in latent AIP, it is supportive rather than diagnostic in isolation and requires genetic confirmation. Renal and hepatic functions were otherwise normal, with a transient mild elevation in creatinine (86 µmol/L; reference range: 45-84 µmol/L for adult women). Plasma porphyrin levels were within normal limits. The relevant porphyria laboratory findings are summarized in Table 1.

**Table 1:** Porphyria-related laboratory findings, reference ranges, and timing of testing relative to symptoms. All samples were obtained during the inter-attack period. Genetic (HMBS) mutation analysis was awaited at the time of reporting. HMBS: hydroxymethylbilane synthase.

Investigation	Result	Reference range	Timing/interpretation
Urinary delta-aminolevulinic acid (ALA)	2.4 µmol/mmol creatinine	<5 µmol/mmol creatinine	Inter-attack period; normal
Urinary porphobilinogen (PBG)	0.8 µmol/mmol creatinine	<1.5 µmol/mmol creatinine	Inter-attack period; normal
Plasma porphyrins	Within normal limits	Within reference range	Inter-attack period; normal
Fecal total porphyrins	72.3 nmol/g	0-49.9 nmol/g	Inter-attack period; elevated
Red cell HMBS activity	38.6 U	Low-normal for the local laboratory	Reduced activity is supportive, not diagnostic; requires genetic confirmation

Following confirmation of elevated fecal porphyrin levels, the patient was reviewed remotely by the specialist service and referred for an outpatient appointment at a UK tertiary porphyria clinic for definitive genetic confirmation and initiation of appropriate management.

## Discussion

The case presented illustrates the diagnostic journey that frequently characterizes AIP, a condition whose clinical manifestations span neurological, gastrointestinal, and psychiatric domains. The initial attribution of the seizures to a psychogenic etiology represents a well-documented diagnostic pitfall. Clinicians may be inclined to diagnose functional or psychiatric disorders when standard investigations are unremarkable, particularly in adolescent women, in whom AIP may not be routinely considered [8]. Although the diagnosis of pseudoseizures was not unreasonable based on the available evidence at the time, it may have contributed to the absence of further metabolic investigation for several years, a pattern reflected in similar case reports and series [9,10].

An important aspect of this case is the timing of biochemical testing in relation to acute attacks. Current diagnostic guidelines recommend urinary PBG as the first-line investigation during a symptomatic episode; however, urinary PBG and ALA levels may normalize following resolution of an acute attack [11]. In this patient, porphyria testing was performed during the inter-attack period, at which time urinary and plasma studies were within normal limits. Clinicians should recognize that normal urinary ALA and PBG levels measured outside an acute attack do not exclude AIP and that these first-line urinary tests are most informative when obtained during a symptomatic episode. This represents a potential source of false reassurance and further diagnostic delay [12].

In this case, the finding that raised biochemical suspicion of acute hepatic porphyria was an elevated fecal porphyrin concentration. This finding may be of particular relevance in AIP, in which fecal coproporphyrins can remain elevated during inter-attack periods and may provide complementary biochemical evidence when urinary and plasma studies are uninformative [13]. Fecal porphyrin analysis, although not universally performed, may therefore be considered a complementary diagnostic investigation in patients with a high clinical suspicion of acute porphyria but nondiagnostic first-line tests. Its interpretation, however, requires consideration and exclusion of other acute hepatic porphyrias and is ideally supported by fractionated porphyrin analysis and genetic testing. The potential role of fecal porphyrin analysis in the diagnostic algorithm warrants broader recognition in clinical guidelines and specialist referral pathways.

The postoperative deterioration in this case is consistent with previous case reports describing perioperative porphyric crises, which may occur within 24-72 hours of surgical intervention in previously undiagnosed patients [8,14]. Metabolic stress and exposure to potentially porphyrogenic anesthetic agents may aggravate the accumulation of porphyrin precursors. This emphasizes the importance of preoperative risk assessment in patients with unexplained recurrent neurovisceral symptoms and the need for tailored anesthetic protocols in suspected or confirmed cases.

Following diagnosis, patients should maintain strict avoidance of potential precipitating factors, including non-prescribed medicines, prolonged fasting, and intercurrent illness. Physicians should provide appropriate family education and facilitate prompt genetic referral to enable cascade screening of at-risk relatives [7].

In summary, the important lesson from this case is that AIP should be included in the differential diagnosis of young women presenting with recurrent unexplained neurovisceral symptoms and inconclusive standard investigations. The absence of the classic biochemical abnormalities during the inter-attack period should not be interpreted as excluding the diagnosis. Fecal porphyrin analysis, if considered at an earlier stage, may have shortened this patient's four-year diagnostic journey and potentially prevented avoidable morbidity [9].

This case report has several limitations. First, genetic confirmation with HMBS mutation analysis was still pending at the time of diagnosis; therefore, the diagnosis is most accurately described as suspected AIP awaiting specialist genetic confirmation. Second, because the case was compiled from medical records spanning four years, several details that would have helped to characterize the acute episodes were not consistently available, including serum sodium concentrations, blood pressure, and heart rate during events. In addition, formal documentation of mental status, objective evidence of motor neuropathy, and the distinction between confirmed epileptic seizures and functional or seizure-like episodes could not be established because of inconclusive electrophysiological studies. Third, the perioperative agents used, including anesthetic, analgesic, antiemetic, and antibiotic medications, as well as the duration of preoperative fasting, could not be reliably retrieved, limiting assessment of potential perioperative triggers. Finally, the coproporphyrin III-to-I ratio and plasma fluorescence scanning were not performed; therefore, the porphyrin pattern could not be fully characterized or differentiated from those of other acute hepatic porphyrias.

## Conclusions

This case highlights how the overlapping neurological, psychiatric, and gastrointestinal manifestations of AIP can lead to prolonged misdiagnosis. Acute porphyria should remain in the differential diagnosis when standard investigations are inconclusive in young women presenting with recurrent neurovisceral symptoms. In the present case, an elevated fecal porphyrin concentration raised suspicion of AIP and prompted specialist referral for genetic confirmation. Early porphyria screening, including fecal porphyrin analysis, may facilitate earlier diagnosis and enable timely specialist referral.
